# Draft Genome Sequence of *Raffaelea quercivora* JCM 11526, a Japanese Oak Wilt Pathogen Associated with the Platypodid Beetle, *Platypus quercivorus*

**DOI:** 10.1128/genomeA.00755-16

**Published:** 2016-07-28

**Authors:** Hayato Masuya, Ri-ichiroh Manabe, Moriya Ohkuma, Rikiya Endoh

**Affiliations:** a Tohoku Research Center, Forestry and Forest Products Research Institute (FFPRI), Morioka, Iwate, Japan; b Genome Network Analysis Support Facility (GeNAS), RIKEN Center for Life Science Technologies, Yokohama, Kanagawa, Japan; c Microbe Division/Japan Collection of Microorganisms (JCM), RIKEN BioResource Center, Tsukuba, Ibaraki, Japan

## Abstract

The Japanese oak wilt pathogen *Raffaelea quercivora* and the platypodid beetle, *Platypus quercivorus*, cause serious mass mortality of *Quercus* spp. in Japan. Here, we present the first draft genome sequence of *R. quercivora* JCM 11526 to increase our understanding of the mechanism of pathogenicity and symbiosis with the ambrosia beetle.

## GENOME ANNOUNCEMENT

The genus *Raffaelea*, belonging to the order *Ophiostomatales*, class *Sordariomycetes*, and phylum *Ascomycota*, is closely associated with various wood-boring beetles in the *Scolytinae* and *Platypodinae* families (1, 2). The fungi strongly depend on the beetles for their dispersal, and the beetle larvae consume the fungi for their own growth (3). A wide variety of platypodid/scolytid beetles possess mycangia, specialized structures for storing fungal spores, on their body or in the mouth (4).

Several *Raffaelea* spp. show virulence toward trees (5, 6). Among them, *R. quercivora* is the causal agent of Japanese oak wilt, together with *Platypus quercivorus* (*Platypodidae*, *Coleoptera*) (7). Since the 1990s, Japanese oak forests have been heavily damaged by the *R. quercivora*/*P. quercivorus* complex. Although virulence tests have been performed in many previous studies (5), they have not been addressed in the context of molecular biology due to a lack of genomic information.

Here, we present the draft genome sequence of *R. quercivora* JCM 11526, an ex-type strain, isolated from an adult *P. quercivorus* and maintained at JCM (7). Genomic DNA of the fungus was extracted with a phenol-chloroform/isoamyl alcohol series, followed by purification using the QIAGEN Genomic-tip 100/G kit (Qiagen) and a PowerClean Pro DNA cleanup kit (Mo Bio Laboratories, Inc.). A paired-end (PE) shotgun library and a mate-pair (MP) library were constructed using the TruSeq DNA sample prep kit (Illumina) and the Nextera mate-pair library prep kit (Illumina); the genomic sequence reads from the PE and MP libraries were generated on the Illumina HiSeq 2500 and MiSeq platforms, respectively. The obtained reads were assembled using ALLPATHS-LG version 52488, where the ploidy was set to 2. The genome was assembled into 33,925,930 bp, with a G+C content of 57.1% in 82 contigs and 26 scaffolds, an *N*_50_ metric of 3,655 kb, and a largest scaffold of 5,014 kb. RepeatMasker (http://www.repeatmasker.org) was used to identify and mask repetitive and low-complex regions within the assembly, and 25% of the genome was estimated to be repetitive. CEGMA (8) was used to assess gene space, resulting in 234 of 248 (94.3%) core eukaryotic genes being identified in the assembly. Gene prediction was performed using the MAKER annotation pipeline (9), including AUGUSTUS version 3.0.3 (10), SNAP (2013-02-16) (11), and GeneMark-ES (12). A total of 8,906 protein-coding genes were predicted using AUGUSTUS trained on *Neurospora crassa*. A total of 214 noncoding RNA genes were predicted using RepeatMasker and RepeatRunner (http://www.yandell-lab.org/software/repeatrunner.html). A total of 126 tRNA genes were predicted using tRNAscan-SE (13). The draft functional annotation was performed using Sma3S (14), where the UniProt-TrEMBL (release 2015_11) and UniProt-SwissProt (release 2015_11) databases were used, resulting in 7,890 and 2,551 genes being functionally annotated by UniProt-TrEMBL and UniProt-SwissProt, respectively.

*R. quercivora* is the first sequenced species in this genus. Information about its genome sequence will provide novel insights into the pathogenicity of *R. quercivora* and the coevolutionary symbiosis between ambrosia beetles, fungi, and host trees.

### Nucleotide sequence accession numbers.

This whole-genome shotgun project has been deposited at DDBJ/ENA/GenBank under the BioProject PRJDB3607 with accession numbers BCFZ01000001 to BCFZ01000026. The version described in this paper is the first one.

## References

[1] Batra LR 1967 Ambrosia fungi: a taxonomic revision, and nutritional studies of some species. Mycologia 59:976–1017. doi:10.2307/3757271.

[2] Massoumi Alamouti S, Tsui CK, Breuil C 2009 Multigene phylogeny of filamentous ambrosia fungi associated with ambrosia and bark beetles. Mycol Res 113:822–835. doi:10.1016/j.mycres.2009.03.003. 19348942

[3] Batra LR 1963 Ecology of ambrosia fungi and their dissemination by beetles. Trans Kans Acad Sci 66:213–236. doi:10.2307/3626562.

[4] Francke-Grosmann H 1967 Ectosymbiosis in wood-inhabiting beetles, p. 141–205. *In* Henry SM (ed), Symbiosis. Academic Press, New York, NY.

[5] Kusumoto D, Masuya H, Hirao T, Goto H, Hamaguchi K, Chou W, Suasa-ard W, Buranapanichpan S, Uraichuen S, Kern-asa O, Sanguansub S, Panmongkol A, Pham QT, Kahono S, Sudiana IM, Kamata N 2015 Comparison of sapwood discoloration in fagaceae trees after inoculation with isolates of *Raffaelea quercivora*, cause of mass mortality of Japanese oak trees. Plant Dis 99:225–230. doi:10.1094/PDIS-06-14-0581-RE. 30699563

[6] Fraedrich SW, Harrington TC, Bates CA, Johnson J, Reid LS, Best GS, Leininger TD, Hawkins TS 2011 Susceptibility to laurel wilt and disease incidence in two rare plant species, pondberry and pondspice. Plant Dis 95:1056–1062. doi:10.1094/PDIS-11-10-0841. 30732063

[7] Kubono T, Ito S 2002 *Raffaelea quercivora* sp. nov. associated with mass mortality of Japanese oak, and the ambrosia beetle (*Platypus quercivorus*). Mycoscience 43:255–260. doi:10.1007/S102670200037.

[8] Parra G, Bradnam K, Korf I 2007 CEGMA: a pipeline to accurately annotate core genes in eukaryotic genomes. Bioinformatics 23:1061–1067. doi:10.1093/bioinformatics/btm071. 17332020

[9] Cantarel BL, Korf I, Robb SM, Parra G, Ross E, Moore B, Holt C, Sánchez Alvarado A, Yandell M 2008 MAKER: an easy-to-use annotation pipeline designed for emerging model organism genomes. Genome Res 18:188–196. doi:10.1101/gr.6743907. 18025269 PMC2134774

[10] Stanke M, Schöffmann O, Morgenstern B, Waack S 2006 Gene prediction in eukaryotes with a generalized hidden Markov model that uses hints from external sources. BMC Bioinformatics 7:62. doi:10.1186/1471-2105-7-62. 16469098 PMC1409804

[11] Johnson AD, Handsaker RE, Pulit SL, Nizzari MM, O’Donnell CJ, de Bakker PI 2008 SNAP: a web-based tool for identification and annotation of proxy SNPs using HapMap. BioInformatics 24:2938–2939. doi:10.1093/bioinformatics/btn564. 18974171 PMC2720775

[12] Ter-Hovhannisyan V, Lomsadze A, Chernoff YO, Borodovsky M 2008 Gene prediction in novel fungal genomes using an ab initio algorithm with unsupervised training. Genome Res 18:1979–1990. doi:10.1101/gr.081612.108. 18757608 PMC2593577

[13] Lowe TM, Eddy SR 1997 tRNAscan-SE: a program for improved detection of transfer RNA genes in genomic sequence. Nucleic Acids Res 25:955–964. doi:10.1093/nar/25.5.0955. 9023104 PMC146525

[14] Muñoz-Mérida A, Viguera E, Claros MG, Trelles O, Pérez-Pulido AJ 2014 Sma3s: a three-step modular annotator for large sequence datasets. DNA Res 21:341–353. doi:10.1093/dnares/dsu001. 24501397 PMC4131829

